# Deucravacitinib in patients with inflammatory bowel disease: 12-week efficacy and safety results from 3 randomized phase 2 studies in Crohn’s disease and ulcerative colitis

**DOI:** 10.1093/ecco-jcc/jjaf080

**Published:** 2025-05-13

**Authors:** Geert D’Haens, Silvio Danese, Remo Panaccione, David T Rubin, Laurent Peyrin-Biroulet, Katsuyoshi Matsuoka, Edward V Loftus, Taku Kobayashi, Walid Elsharkawi, Rosa Miceli, Samia Ahmed, Yi Luo, Andrew Napoli, John Vaile, Quentin Dornic, Aditya Patel, Stefan Schreiber

**Affiliations:** Department of Gastroenterology and Hepatology, Amsterdam University Medical Centers, Amsterdam, The Netherlands; Department of Gastroenterology and Endoscopy, IRCCS Ospedale San Raffaele and Vita-Salute San Raffaele University, Milan, Italy; Department of Medicine, Inflammatory Bowel Disease Clinic, Calgary, Alberta, Canada; Inflammatory Bowel Disease Center, University of Chicago Medicine, Chicago, IL, United States; Department of Gastroenterology, INFINY Institute, INSERM NGERE, CHRU Nancy, Vandœuvre-lès-Nancy, France; Groupe Hospitalier Privé Ambroise Paré - Hartmann, Paris IBD Center, Neuilly sur Seine, France; Division of Gastroenterology and Hepatology, McGill University Health Centre, Montreal, Quebec, Canada; Division of Gastroenterology and Hepatology, Toho University Sakura Medical Center, Sakura, Chiba, Japan; Division of Gastroenterology and Hepatology, Mayo Clinic College of Medicine and Science, Rochester, MN, United States; Kitasato University Kitasato Institute Hospital, Tokyo, Japan; Bristol Myers Squibb, Princeton, NJ, United States; Bristol Myers Squibb, Princeton, NJ, United States; Bristol Myers Squibb, Princeton, NJ, United States; Bristol Myers Squibb, Princeton, NJ, United States; Bristol Myers Squibb, Princeton, NJ, United States; Bristol Myers Squibb, Princeton, NJ, United States; Bristol Myers Squibb, Princeton, NJ, United States; Bristol Myers Squibb, Princeton, NJ, United States; Department of Internal Medicine I, University Hospital Schleswig-Holstein, Kiel University, Kiel, Germany

**Keywords:** Deucravacitinib, Crohn’s disease, ulcerative colitis, efficiacy, safety

## Abstract

**Background and Aims:**

Tyrosine kinase 2 is a downstream intracellular mediator of interleukin-23 signaling, which has a key role in the pathogenesis of inflammatory bowel disease. Deucravacitinib is a novel, oral, selective, allosteric tyrosine kinase 2 inhibitor currently approved for the treatment of adults with moderate to severe plaque psoriasis.

**Methods:**

Here we describe 3 randomized, double-blind, placebo-controlled phase 2 studies of deucravacitinib in patients with moderately to severely active Crohn’s disease (LATTICE-CD [NCT03599622]) or ulcerative colitis (LATTICE-UC [NCT03934216] and IM011-127 [NCT04613518]). Patients were randomized to receive placebo or twice-daily deucravacitinib 3 or 6 mg (LATTICE-CD), 6 mg (LATTICE-UC), or 12 mg (IM011-127) for 12 weeks. Coprimary endpoints for LATTICE-CD were clinical remission and endoscopic response at week 12. The primary endpoint was clinical remission (per modified Mayo score) at week 12 for LATTICE-UC and clinical response (per modified Mayo score) at week 12 for IM011-127.

**Results:**

A total of 239 (LATTICE-CD), 131 (LATTICE-UC), and 38 (IM011-127) patients were randomized. The primary endpoints were not met for all 3 studies, which resulted in early study termination for LATTICE-CD and IM011-127. High efficacy rates were observed in placebo groups throughout the studies. In all studies, the safety profile of deucravacitinib was consistent with the known safety profile observed in patients with psoriasis, and no new safety signals were observed.

**Conclusions:**

Deucravacitinib at multiple doses did not demonstrate significant clinical benefit vs placebo in moderately to severely active Crohn’s disease or ulcerative colitis. Deucravacitinib was safe and well tolerated.

## 1. Introduction

Inflammatory bowel disease (IBD), which consists of Crohn’s disease (CD) and ulcerative colitis (UC), is characterized by a chronic, dysregulated inflammatory response within the gastrointestinal tract.^1,2^ Causes of IBD are multifactorial and include genetic, environmental, immunologic, and lifestyle factors, resulting in significant heterogeneity of the disease.^2,3^ Despite treatment advances for IBD, current biologic treatment options have been able to achieve overall remission rates of only 18%–48% depending on the specific biologic treatment.^4^ Thus, novel therapeutic options are needed due to initial nonresponse or loss of response over time observed with most therapies.^5–7^

Interleukin 23 (IL-23) is a key cytokine in IBD pathogenesis^8–10^ and targeting IL-23 has been successful as a therapeutic strategy.^11^ However, IL-23 inhibitors currently approved for IBD are biologics with parenteral routes of administration, which may be unfavorable or inconvenient for patients.^12–14^

Tyrosine kinase 2 (TYK2) is a downstream intracellular mediator of multiple cytokines, including IL-23 and IL-12, but not IL-2 signaling, which may be protective in UC.^15–19^ TYK2 inhibition has been shown to be effective in other immune-mediated inflammatory diseases such as psoriasis and psoriatic arthritis, for which therapies with anti-IL-23 monoclonal antibodies are highly effective.^17,20^ Additionally, a previous animal model demonstrated that inhibition of TYK2 signaling in T cells reduced naive CD4 T-cell differentiation into Th1 and Th17 cells and ameliorated colitis pathogenesis.^18^ As a result, inhibition of TYK2 is a target of interest for IBD.

Deucravacitinib is a novel, oral, selective, allosteric TYK2 inhibitor currently approved for the treatment of adults with moderate to severe plaque psoriasis.^17,21^ The allosteric inhibition makes deucravacitinib highly selective for TYK2 vs members of the JAK family (ie, JAK1, JAK2, and JAK3).^22^ This approach differentiates deucravacitinib from nonselective inhibitors of the JAK family of kinases that target the highly conserved active site of the kinase domain.^22^ To date, the safety profile of deucravacitinib is differentiated from JAK inhibitors, without evidence of an increased risk of major adverse cardiovascular events, venous thromboembolism, malignancy, or meaningful changes in laboratory parameters, and is attributed to the high selectivity of deucravacitinib for TYK2.^17,22–24^ Deucravacitinib demonstrated efficacy in mouse models of IBD by inhibiting autoimmune effects (ie, prevented disease-associated weight loss in mice with systemic wasting disease driven by IL-12 and IL-23 with protection against histologically evident colitis).^25^ As a result, deucravacitinib was investigated for the treatment of IBD in clinical trials.^26^ The deucravacitinib phase 2 development program for IBD consisted of 3 randomized, double-blind, placebo-controlled phase 2 studies that assessed the efficacy and safety of deucravacitinib in patients with moderately to severely active CD (LATTICE-CD [NCT03599622]^27^), or UC (LATTICE-UC [NCT03934216]^28,29^ and IM011-127 [NCT04613518]^30^). This manuscript reports the efficacy and safety of deucravacitinib during the induction phases of these 3 trials, which were the first trials performed with a TYK2 inhibitor in patients with UC or CD.

## 2. Methods

Trial protocols and statistical analysis plans are published on clinicaltrials.gov: LATTICE-CD (Study Details | An Investigational Study of Experimental Medication BMS-986165 in Participants With Moderate to Severe Crohn’s Disease | ClinicalTrials.gov), LATTICE-UC (Study Details | Safety and Efficacy of Deucravacitinib in Participants With Moderate to Severe Ulcerative Colitis | ClinicalTrials.gov), and IM011-127 (Study Details | A Study of the Safety, Efficacy, and Biomarker Response of BMS-986165 in Participants With Moderate to Severe Ulcerative Colitis | ClinicalTrials.gov).

### 2.1. Study design

#### 2.1.1. LATTICE-CD

**Figure 1.**
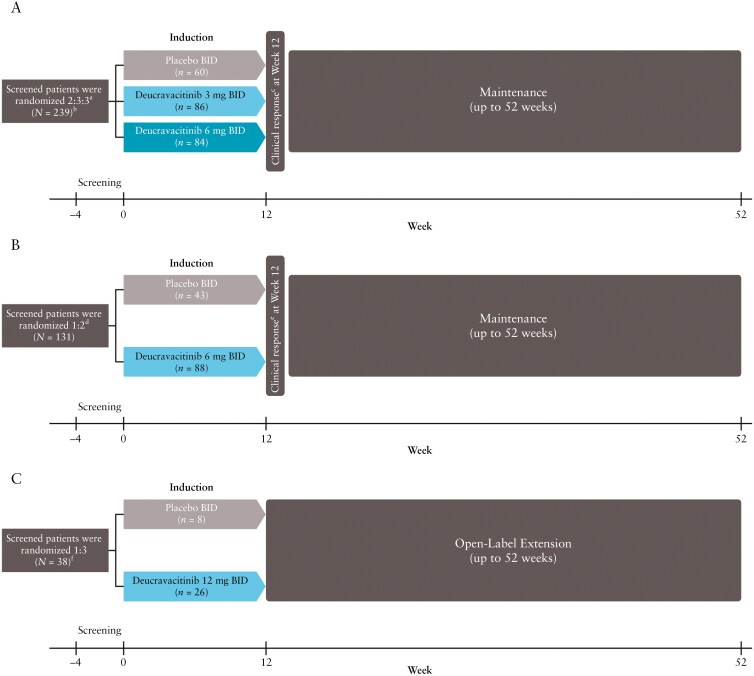
Study designs for the 12-week induction period of LATTICE-CD [A], LATTICE-UC [B], and IM011-127 [C]. ^a^Patients were stratified by geographic region (United States, Japan, rest of world), prior anti-TNF exposure, and concomitant corticosteroid use (yes or no). ^b^Nine patients were initially randomized to the additional once-daily deucravacitinib 12-mg treatment arm, which was removed for adequate powering of the study. ^c^Defined as a reduction from baseline in the Crohn’s Disease Activity Index (CDAI) of ≥100 points or a total CDAI of <150). ^d^Patients were stratified by prior biologic exposure (includes Janus kinase inhibitors; no prior exposure, exposure to 1 biologic, or exposure to more than 1 biologic) and corticosteroid use (yes or no). ^e^Defined as a reduction from baseline of ≥1 point or absolute score of ≤1 point in rectal bleeding subscore plus a reduction of ≥2 points and ≥35% on the 3-component Mayo score or ≥3 points and ≥30% on the 4-component Mayo score. ^f^Four patients were initially randomized to an additional 6-mg BID deucravacitinib treatment group, which was removed due to the negative efficacy results observed with this dose in LATTICE-UC. BID, twice daily; CD, Crohn’s disease; TNF, tumor necrosis factor; UC, ulcerative colitis.

In LATTICE-CD, eligible patients were randomized in a blinded fashion in a 2:3:3 ratio to receive placebo or twice-daily (BID) oral deucravacitinib 3 or 6 mg, respectively, for 12 weeks in the induction period (Figure 1A). A few patients (*N* = 9) were initially randomized to an additional treatment arm of once-daily oral deucravacitinib 12 mg; however, this treatment arm was later removed to allow for increased powering of the other dose arms and because pharmacokinetic results of an early interim analysis demonstrated undifferentiated exposure between the once-daily and twice-daily dosing arms. Results for the once-daily deucravacitinib 12-mg treatment arm were not included in efficacy analyses but were included in safety analyses. Randomization was stratified by geographic region (United States, Japan, rest of the world), prior exposure to antitumor necrosis factor agents (yes or no), and concomitant corticosteroid use (yes or no).

#### 2.1.2. LATTICE-UC

In LATTICE-UC, eligible patients were randomized in a blinded fashion in a 1:2 ratio to receive placebo or oral deucravacitinib 6 mg BID, respectively, for 12 weeks in the induction period (Figure 1B). Randomization was stratified by corticosteroid use (yes or no) and prior exposure to biologics, including JAK inhibitors, which are indicated for the treatment of UC (no prior exposure [0], exposure to one biologic [1], or exposure to more than one biologic [>1]). Progression into the maintenance period was similar to LATTICE-CD.

#### 2.1.3. IM011-127

The IM011-127 study was initiated to evaluate whether a higher dose of deucravacitinib was effective. In IM011-127, eligible patients were randomized in a blinded fashion in a 1:3 ratio to receive placebo or oral deucravacitinib 12 mg BID, respectively, in the 12-week induction period (Figure 1C). A small number of patients (*N* = 4) were initially randomized to an additional treatment arm of oral deucravacitinib 6 mg BID; however, this treatment arm was later removed due to failure to meet week-12 induction efficacy endpoints with this dose in the LATTICE-UC study. Therefore, the current analysis includes efficacy and safety outcomes for the deucravacitinib 12-mg BID group in IM011-127.

Assessed dosages for deucravacitinib varied throughout the deucravacitinib IBD clinical program because doses were based on preclinical animal models of efficacy and clinical pharmacokinetics, target engagement, efficacy, and safety assessments.

This report includes only results from the placebo-controlled 12-week induction periods from LATTICE-CD, LATTICE-UC, and IM011-127 because some trials were terminated early.

### 2.2. Patients

#### 2.2.1. LATTICE-CD

Patients 18–75 years of age, inclusive, with ≥3 months of moderately to severely active CD with ileal, colonic, or ileocolonic disease distribution were enrolled. Moderate to severe CD was defined as having all 3 of the following: a Crohn’s Disease Activity Index (CDAI) score of 220–450; a patient-reported outcome based on the stool frequency and abdominal pain components of the CDAI (PRO2), with an average daily score for abdominal pain ≥2 or average daily number of very soft (loose) or liquid (watery) stools (Bristol Stool Scale type 6 or 7 only) ≥4; and evidence of active inflammation in at least 1 of the 5 ileocolonic segments (based on central reading) with a total Simple Endoscopic Score for CD (SES-CD) of ≥6 or an SES-CD of ≥4 if only isolated ileitis was found with baseline endoscopy. Patients were also required to have an inadequate response, loss of response, or intolerance to one or more standard treatment courses for CD (ie, 5-aminosalicylate acids [5-ASAs], corticosteroids, immunomodulators, biologics). Doses of previous therapies with 5-ASAs or oral corticosteroids (≤20-mg once-daily prednisone or equivalent, ≤9-mg once-daily budesonide or equivalent) must have been stable for ≥2 weeks before randomization. Corticosteroid doses could not have been increased above the baseline dose or newly initiated in those who did not enter the study on stable corticosteroids. If disease worsening occurred (ie, flares) and rescue corticosteroids were required, patients must have discontinued the study treatment to receive appropriate alternative available treatment. Concomitant immunomodulator therapy was not permitted during the study. Additional inclusion and exclusion criteria are listed in the Supplementary Methods.

#### 2.2.2. LATTICE-UC

LATTICE-UC enrolled patients 18–80 years of age inclusive with moderately to severely active UC for ≥3 months, defined as a modified Mayo score of 5–9 points, which consists of a stool frequency subscore of ≥2, rectal bleeding subscore of ≥1, and a screening Mayo endoscopic subscore (MES) of ≥2. Patients had an inadequate response, loss of response, or intolerance to 1 or more standard (eg, 5-ASAs, corticosteroids, immunomodulators) or biologic (ie, anti-TNFs, integrin inhibitors, anti-IL-12/IL-23p40 antibodies) treatments for UC. Use of concomitant 5-ASAs or oral corticosteroids (≤20 mg once-daily prednisone or equivalent, ≤9 mg once-daily budesonide or equivalent) was permitted, provided patients were at a stable dose for ≥2 weeks before randomization and maintained this dose during the induction period. The same restrictions regarding dose modification or initiation of corticosteroids described for LATTICE-CD were also applicable for LATTICE-UC. The exclusion criteria for LATTICE-UC were generally similar to those for LATTICE-CD in the Supplementary Methods, except that patients with failure of or loss of response to JAK inhibitors were excluded from LATTICE-UC. Patients could not take concomitant immunomodulators during the study.

#### 2.2.3. IM011-127

Key inclusion criteria were similar to those for LATTICE-UC except for patient age and prior corticosteroid treatment. In IM011-127, patients were 18–65 years of age inclusive with 1 or more prior treatment course of corticosteroids for UC within a 4-week induction regimen (i.e. oral prednisone ≥30 mg/day or equivalent for ≥2 weeks or oral budesonide 9 mg/day or equivalent for ≥4 weeks). Use of concomitant 5-ASAs or oral corticosteroids (≤20 mg once-daily prednisone or equivalent, ≤9 mg once-daily budesonide or equivalent) was permitted, provided patients were at a stable dose for ≥2 weeks before randomization and maintained this dose during the induction period. The restrictions described for the other trials regarding dose modification or initiation of corticosteroids during the study applied to this study as well. The exclusion criteria for IM011-127 were generally similar to those for LATTICE-UC. Patients were not allowed to be on concomitant immunomodulators.

### 2.3. Efficacy outcomes

All efficacy outcomes were assessed in the overall randomized population. Definitions for all efficacy outcomes presented in this analysis are included in Table S1. Endoscopy video recordings and biopsy samples were centrally read.

#### 2.3.1. LATTICE-CD

Coprimary efficacy endpoints were the proportion of patients who achieved clinical remission and the proportion of patients who achieved endoscopic response at week 12. These efficacy endpoints were also assessed by prior biologic exposure (biologic naive vs biologic exposed). Secondary endpoints were the proportions of patients who achieved clinical response and PRO2 remission at week 12 and the mean change from baseline in SES-CD at week 12.

#### 2.3.2. LATTICE-UC

The primary efficacy endpoint was the proportion of patients who achieved clinical remission (modified Mayo score) at week 12. Secondary endpoints were clinical response, endoscopic response, and histologic improvement at week 12. The secondary endpoints presented in this study were the proportions of patients who achieved clinical response and endoscopic response at week 12. Efficacy outcomes were also assessed by prior biologic exposure (biologic naive vs biologic exposed [exposure to 1 or more biologic] as categorized by the randomization stratification factor, which included JAK inhibitors).

#### 2.3.3. IM011-127

The primary efficacy endpoint was the proportion of patients in clinical response (modified Mayo score) at week 12. Exploratory endpoints evaluated in this analysis were the proportions of patients in clinical remission (modified Mayo score), with endoscopic improvement, and in endoscopic remission at week 12.

### 2.4. Safety outcomes

For all trials, safety was assessed by adverse events (AEs), serious AEs (SAEs), AEs leading to discontinuation, and changes from baseline in clinical laboratory results that occurred during the 12-week induction period. AEs were reported by patients. Investigators were responsible for detecting, documenting, and reporting events that met the definition of an AE or SAE. An external data monitoring committee monitored safety by blinded treatment group.

### 2.5. Statistical analyses

All efficacy outcomes for the 3 studies were analyzed in the intention-to-treat population, which included all patients who were randomized to receive a study treatment. For all studies, nonresponder imputation (NRI) was the primary method of imputation for the coprimary or primary efficacy endpoints, which accounts for missing endpoint data.

Safety outcomes were analyzed in all patients who received at least 1 dose of double-blind study treatment, and safety results are summarized descriptively.

#### 2.5.1. LATTICE-CD

Coprimary endpoints were analyzed using a stratified Cochran–Mantel–Haenszel (CMH) test stratified by factors used for randomization stratification to compare response rates between each deucravacitinib treatment group and placebo.

Binary secondary endpoints were analyzed using a CMH test stratified by factors used for randomization stratification to compare the response rates between each deucravacitinib group and the placebo group. Analysis of covariance, with treatment and randomization stratification factors as fixed effects, was used for continuous secondary endpoints. An NRI analysis was used for binary secondary efficacy endpoints, and multiple imputation analysis was used for continuous secondary endpoints, which both accounted for missing endpoint data. A Bonferroni adjustment was used to compare the deucravacitinib treatment groups with the placebo group.

For all endpoints, differences in response rates and corresponding two-sided 95% confidence intervals (CIs) were described along with point estimates and corresponding 95% CIs for each treatment group using a binomial method.

#### 2.5.2. LATTICE-UC

The primary efficacy endpoint of clinical remission at week 12 was analyzed using a stratified CMH test, and comparisons between deucravacitinib 6 mg BID and placebo were adjusted for the randomization stratification factors. Secondary efficacy endpoints were analyzed in a hierarchical fashion to control for type 1 error rate inflation, in which the primary efficacy endpoint determined testing progression for the secondary efficacy endpoints. Each secondary efficacy endpoint was tested sequentially in a fixed-sequence order of clinical response, endoscopic response, and histologic improvement at week 12 using a 1-sided alpha value of 0.1 for significance testing. No further testing was performed once a test failed to show significance. Similarly to the primary efficacy endpoint, NRI analyses were utilized. Efficacy endpoints analyzed by prior biologic exposure were not further stratified based on the factors used for randomization.

#### 2.5.3. IM011-127

The primary efficacy endpoint of clinical response at week 12 was estimated using the Clopper–Pearson exact method.

Additional details regarding statistical analyses can be found in the Supplementary Methods.

### 2.6. Ethics

This analysis adhered to the Good Clinical Practices guidelines and the ethical principles outlined in the Declaration of Helsinki. Investigators at each study site obtained protocol and informed consent approval by an institutional review board or independent ethics committee. Written consent was obtained from each patient in the study, which was sponsored by Bristol Myers Squibb.

## 3. Results

### 3.1. Patients

#### 3.1.1. LATTICE-CD

**Figure 2.**
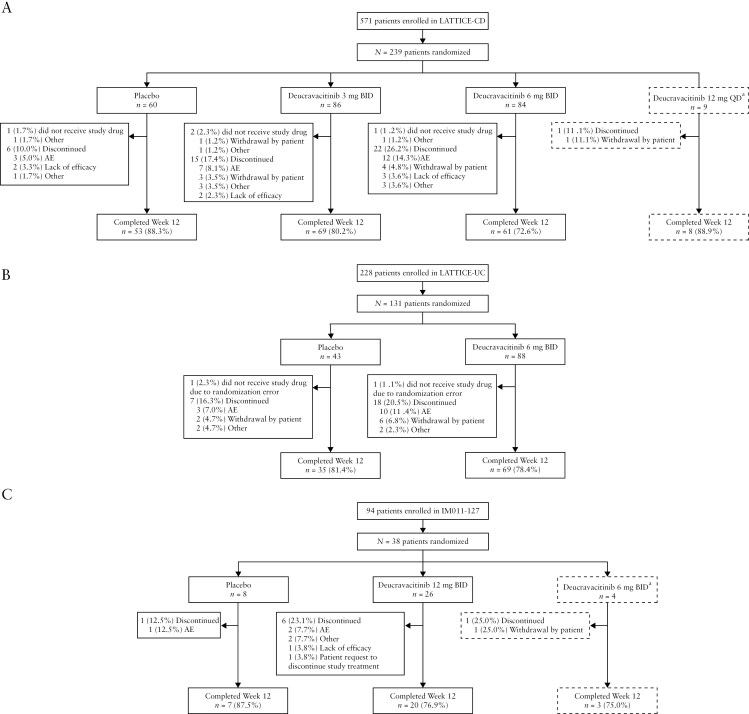
Patient disposition through the induction period (0–12 weeks) of LATTICE-CD (A), LATTICE-UC (B), and IM011-127 (C) (intention-to-treat population). ^a^Treatment was discontinued. AE, adverse event; BID, twice daily; CD, Crohn’s disease; QD, once daily; UC, ulcerative colitis.

In LATTICE-CD, 571 patients were enrolled and screened, of whom 239 patients were randomized to receive placebo (*n* = 60), deucravacitinib 3 mg BID (*n* = 86), or deucravacitinib 6 mg BID (*n* = 84) (Figure 2A). The number of patients enrolled and randomized by country and the list of investigators by country are reported in Tables S2 and S3, respectively. Of those randomized, 59, 84, and 83 patients were treated with placebo, deucravacitinib 3 mg BID, or deucravacitinib 6 mg BID, respectively. Overall, 53, 69, and 61 patients in the placebo, deucravacitinib 3-mg BID, and deucravacitinib 6-mg BID groups completed the induction period. The most common reason for treatment discontinuation was AEs (5.0% in the placebo group, 8.1% in the deucravacitinib 3-mg BID group, and 14.3% in the deucravacitinib 6-mg BID group). Of the AEs that led to treatment discontinuation, CD was the most frequent in both deucravacitinib groups. This study was terminated early during the maintenance period since the primary endpoints in the 12-week induction period were not met.

#### 3.1.2. LATTICE-UC

In LATTICE-UC, 228 patients were enrolled and screened, and 131 of these patients were randomized to receive placebo (*n* = 43) or deucravacitinib 6 mg BID (*n* = 88) (Figure 2B). The number of patients enrolled and randomized by country and the list of investigators by country are reported in Tables S2 and S3. All except 1 patient in each treatment group received the randomized treatment. A total of 35 and 69 patients in the placebo and deucravacitinib 6-mg BID groups completed the 12-week induction period, respectively. The most common reason for treatment discontinuation for both the placebo and deucravacitinib groups was AEs (7.0% and 11.4%, respectively).

#### 3.1.3. IM011-127

In IM011-127, 94 patients were enrolled and screened, of whom 38 patients were randomized to receive placebo (*n *= 8) or deucravacitinib 12 mg BID (*n *= 26) (Figure 2C; 4 patients were randomized to deucravacitinib 6 mg BID, which was later excluded from the study as described in the Methods). The number of patients enrolled and randomized by country and the list of investigators by country are reported in Tables S2 and S3. All randomized patients received study treatment. The most common reason for treatment discontinuation in the placebo and deucravacitinib 12-mg BID groups was AEs (12.5% and 7.7%, respectively). This study was terminated early since the primary endpoint in the 12-week induction period was not met.

### 3.2. Baseline characteristics

Baseline characteristics were generally balanced across treatment groups in all studies (Tables 1 and 2); however, a few differences between the treatment groups were observed. In LATTICE-CD, slightly more patients in the deucravacitinib 3-mg BID group vs deucravacitinib 6-mg BID and placebo groups were Hispanic or Latino (8.1% vs 2.4% and 3.3%, respectively). Additionally, the deucravacitinib 6-mg BID group versus the deucravacitinib 3-mg BID and placebo groups had a higher proportion of patients with ileum-only disease (23.8% vs 20.9% and 15.0%, respectively) and a higher mean CDAI (346.9 vs 329.5 and 325.1, respectively) at baseline. In LATTICE-UC, a slightly lower percentage of patients in the deucravacitinib 6-mg BID versus placebo group had a baseline modified Mayo score ≤7 (64.8% vs 72.1%, respectively). Similarly, in IM011-127, patients in the placebo group had a slightly lower mean modified Mayo score at baseline compared with that of the deucravacitinib 12-mg BID group (6.5 vs 7.1, respectively). In LATTICE-UC, additional differences in baseline characteristics between the deucravacitinib 6-mg BID and placebo groups were observed in patient weight ≥90 kg (16.3% and 25.0%, respectively) and median MES (3.0 and 2.0, respectively).

**Table 1.** Baseline demographics and clinical characteristics in the induction period of LATTICE-CD.

Characteristics	Placebo, *n* = 60	Deucravacitinib, 3 mg BID, *n* = 86	Deucravacitinib, 6 mg BID, *n* = 84
Sex, male, *n* (%)	38 (63.3)	48 (55.8)	50 (59.5)
Age, years, mean (SD)	39.1 (16.7)	39.5 (15.2)	37.9 (14.6)
≥65 years, *n* (%)	8 (13.3)	5 (5.8)	4 (4.8)
Race, White, *n* (%)	48 (80.0)	70 (81.4)	68 (81.0)
Ethnicity, Hispanic or Latino, *n* (%)	2 (3.3)	7 (8.1)	2 (2.4)
Baseline weight, kg, mean (SD)	73.6 (20.7)	75.6 (24.3)	70.3 (16.9)
≥90 kg, *n* (%)	14 (23.3)	21 (24.4)	11 (13.1)
Baseline BMI, kg/m^2^, mean (median)	24.8 (23.2)	25.3 (23.6)	23.9 (23.8)
Geographic region, *n* (%)			
Rest of world	39 (65.0)	53 (61.6)	53 (63.1)
United States	16 (26.7)	26 (30.2)	24 (28.6)
Japan	5 (8.3)	7 (8.1)	7 (8.3)
Smoking status, *n* (%)			
Currently nonsmoker	53 (88.3)	67 (77.9)	69 (82.1)
Smoker	7 (11.7)	19 (22.1)	15 (17.9)
Duration of disease, ≥10 years, *n* (%)	26 (43.3)	44 (51.2)	33 (39.3)
Age at disease onset, *n* (%)			
<18 years	16 (26.7)	24 (27.9)	21 (25.0)
18–39 years	34 (56.7)	44 (51.2)	50 (59.5)
≥40 years	8 (13.3)	18 (20.9)	13 (15.5)
Not reported	2 (3.3)	0 (0.0)	0 (0.0)
Disease distribution, *n* (%)			
Ileum only	9 (15.0)	18 (20.9)	20 (23.8)
Colon only	14 (23.3)	18 (20.9)	16 (19.0)
Ileum and colon	37 (61.7)	50 (58.1)	48 (57.1)
Perianal disease, *n* (%)	21 (35.0)	21 (24.4)	20 (23.8)
Upper GI disease, *n* (%)	8 (13.3)	18 (20.9)	17 (20.2)
Baseline corticosteroid use,^a^ *n* (%)	22 (36.7)	34 (39.5)	30 (35.7)
Prior exposure to biologics, *n* (%)			
Yes	41 (68.3)	53 (61.6)	57 (67.9)
No	19 (31.7)	33 (38.4)	27 (32.1)
Prior exposure to anti-TNF agent,^a^ *n* (%)	36 (60.0)	52 (60.5)	53 (63.1)
Baseline CDAI, mean (SD)	325.1 (86.5)	329.5 (88.8)	346.9 (96.4)
Baseline SES-CD, mean (SD)	13.4 (6.3)	12.2 (6.2)	13.2 (6.9)

Abbreviations: BID, twice daily; BMI, body mass index; CD, Crohn’s disease; CDAI, Crohn’s Disease Activity Index; GI, gastrointestinal; SD, standard deviation; SES-CD, Simple Endoscopic Score for Crohn’s Disease; TNF, tumor necrosis factor.

^a^Based on case report forms.

**Table 2.** Baseline demographics and clinical characteristics in the induction period of LATTICE-UC and IM011-127.

Characteristics	LATTICE-UC		IM011-127	
	Placebo, *n* = 43	Deucravacitinib, 6 mg BID *n* = 88	Placebo, *n* = 8	Deucravacitinib, 12 mg BID *n *= 26
Sex, male, *n* (%)	29 (67.4)	48 (54.5)	6 (75.0)	14 (53.8)
Age, years, mean (SD)	40.3 (13.9)	41.6 (14.8)	38.0 (15.1)	41.8 (14.4)
≥65 years, *n* (%)	4 (9.3)	7 (8.0)	NA	NA
Race, White, *n* (%)	34 (79.1)	80 (90.9)	7 (87.5)	25 (96.2)
Baseline weight, kg, mean (SD)	75.8 (19.0)	76.6 (17.4)	75.2 (18.8)	77.7 (19.1)
≥90 kg, *n* (%)	22 (25.0)	7 (16.3)	1 (12.5)	7 (26.9)
Baseline BMI, kg/m^2^, mean (SD)	25.1 (5.9)	26.1 (5.7)	24.0 (4.5)	26.2 (5.0)
Geographic region, *n* (%)^a^				
United States	6 (14.0)	18 (20.5)	NA	NA
Japan	4 (9.3)	2 (2.3)	NA	NA
Rest of world	33 (76.7)	68 (77.3)	0 (0.0)	0 (0.0)
Europe	NA	NA	7 (87.5)	19 (73.1)
North America	NA	NA	1 (12.5)	7 (26.9)
Smoking status, *n* (%)				
Currently nonsmoker	38 (88.4)	82 (93.2)	8 (100.0)	25 (96.2)
Smoker	5 (11.6)	6 (6.8)	0 (0.0)	1 (3.8)
Duration of disease, years, mean (SD)	7.6 (7.3)	8.4 (6.6)	8.1 (7.0)	8.3 (9.3)
Age at disease diagnosis, years, mean (SD)	33.4 (13.4)	33.9 (13.9)	30.4 (11.4)	34.1 (13.3)
Disease distribution, *n* (%)				
Proctosigmoiditis	10 (23.3)	28 (31.8)	0 (0.0)	0 (0.0)
Left-sided colitis	19 (44.2)	33 (37.5)	4 (50.0)	20 (76.9)
Extensive colitis	14 (32.6)	27 (30.7)	4 (50.0)	6 (23.1)
Concomitant corticosteroid use, *n* (%)	18 (41.9)	35 (39.8)	5 (62.5)	13 (50.0)
Prior exposure to biologics, *n* (%)				
No exposure	27 (62.8)	56 (63.6)	4 (50.0)	21 (80.8)
Exposure to 1	9 (20.9)	19 (21.6)	0 (0.0)	3 (11.5)
Exposure to >1	7 (16.3)	13 (14.8)	4 (50.0)	2 (7.7)
Modified Mayo score,^b^ mean (SD)	6.7 (1.1)	6.9 (1.0)	6.5 (1.2)	7.1 (1.1)
≤7	31 (72.1)	57 (64.8)	NA	NA
>7	11 (25.6)	27 (30.7)		
Not reported	1 (2.3)	4 (4.5)		
MES				
Mean (SD)	2.4 (0.5)	2.7 (0.5)	2.6 (0.5)	2.6 (0.5)
Median (min, max)	2.0 (1, 3)	3.0 (2, 3)	3.0 (2, 3)	3.0 (2, 3)

Abbreviations: BID, twice daily; BMI, body mass index; max, maximum; MES, Mayo endoscopic subscore; min, minimum; NA, not applicable; RBS, rectal bleeding subscore; SD, standard deviation; SFS, stool frequency subscore; UC, ulcerative colitis.

^a^Patients’ geographic regions were categorized as United States, Japan, or rest of world in LATTICE-UC and as Europe, North America, or rest of world in IM011-127.

^b^The modified Mayo score (0–9 points) is the sum of the SFS, RBS, and MES (each individual subscore ranges from 0 to 3 points).

### 3.3. Efficacy

#### 3.3.1. LATTICE-CD

**Figure 3.**
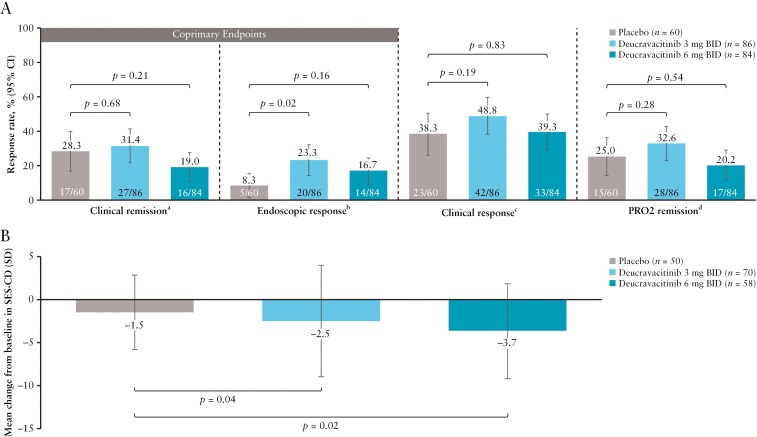
Week 12 efficacy in LATTICE-CD in clinical, endoscopic, and PRO2 outcomes (A) and changes from baseline in SES-CD (ANCOVA [MI]) (intention-to-treat population) (B). ^a^Defined as CDAI of <150. ^b^Defined as ≥50% improvement from baseline in SES-CD. ^c^Defined as reduction from baseline in the CDAI of ≥100 points or a total CDAI of <150. ^d^Defined as an unweighted average daily score for abdominal pain ≤1 and average number of BSS type 6 or 7 stools ≤3 on the PRO2 and both not worse than baseline. ANCOVA, analysis of covariance; BID, twice daily; BSS, Bristol Stool Scale; CI, confidence interval; CDAI, Crohn’s Disease Activity Index; MI, multiple imputation; PRO2, patient-reported outcome based on the stool frequency and abdominal pain components of the CDAI; SD, standard deviation; SES-CD, Simple Endoscopic Score for Crohn’s Disease.

The study did not meet its coprimary endpoints of clinical remission and endoscopic response at week 12 (Figure 3A). No statistically significant improvements in clinical remission rates between the deucravacitinib 3-mg and 6-mg BID groups versus the placebo group were observed (31.4% [*P* = .68] and 19.0% [*P* = .21] vs 28.3%, respectively).

The endoscopic response rate in the deucravacitinib 3-mg BID group (23.3%) was significantly higher than that of the placebo group (8.3%; *P *= .02). Endoscopic response was achieved in 16.7% versus 8.3% (*P* = .16) of patients in the deucravacitinib 6-mg BID group versus placebo group, respectively.

Clinical response rates were achieved in 48.8%, 39.3%, and 38.3% of patients in the deucravacitinib 3-mg BID group, deucravacitinib, 6-mg BID group, and the placebo group, respectively; however, improvements were not statistically significant between the deucravacitinib 3-mg and 6-mg BID groups versus placebo (*P *= .19 and *P *= .83, respectively) (Figure 3A). Additionally, PRO2 remission rates were not significantly different between the deucravacitinib 3-mg and 6-mg BID groups vs the placebo group (32.6% [*P* = .28] and 20.2% [*P* = .54] vs 25.0%, respectively) (Figure 3A). Decreases from baseline in SES-CD scores at week 12 were observed in all treatment groups, with significantly greater mean changes from baseline observed in the deucravacitinib 3-mg and 6-mg BID groups vs the placebo group (−2.5 and −3.7 vs −1.5; *P* = .04 and *P* = .02, respectively) (Figure 3B).

In biologic-naive patients, clinical remission was achieved in 42.1% of patients in the placebo versus 33.3% (*P* = .53) in the deucravacitinib 3-mg and 29.6% (*P* = .39) in the 6-mg BID groups; patients receiving deucravacitinib 3 mg BID versus placebo achieved a clinical remission rate of 30.2% versus 22.0% (*P* = .37) in biologic-exposed patients (Figure S1). An endoscopic response rate of 33.3% versus 15.8% (*P* = .17) was achieved in the deucravacitinib 3-mg BID versus the placebo groups, respectively, in biologic-naive patients; 17.0%, 17.5%, and 4.9% of biologic-exposed patients achieved endoscopic response in the deucravacitinib 3-mg BID, deucravacitinib 6-mg BID, and the placebo groups, respectively.

#### 3.3.2. LATTICE-UC

**Figure 4.**
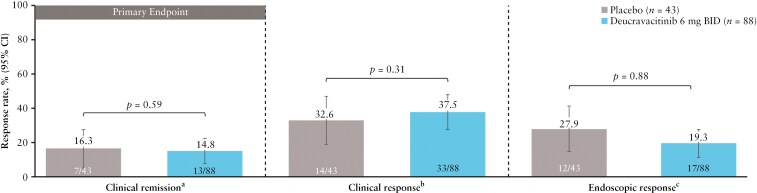
Week 12 efficacy in LATTICE-UC (intention-to-treat population). ^a^Defined as an SFS of ≤1 with ≥1-point decrease from baseline, RBS of 0, and MES of ≤1 without friability. ^b^Reduction in the modified Mayo score of ≥2 points and ≥30% from baseline and a decrease in the RBS of ≥1 point or an absolute RBS of ≤1. The Mayo score (0–9 points) is the sum of the SFS, RBS, and MES (each individual subscore ranges from 0 to 3 points). ^c^Defined as an MES of ≤1. BID, twice daily; CI, confidence interval; MES, Mayo endoscopic subscore; RBS, rectal bleeding subscore; SFS, stool frequency subscore.

The study did not meet its primary endpoint of clinical remission at week 12. Rates of clinical remission at week 12 were similar in the deucravacitinib 6-mg BID group and the placebo group (14.8% vs 16.3% [*P* = .59], respectively). Clinical response at week 12 was achieved in 37.5% vs 32.6% (*P* = .31) of patients in the deucravacitinib 6-mg BID group versus the placebo group (Figure 4). Endoscopic response at week 12 was achieved in 19.3% of patients in the deucravacitinib 6-mg BID group versus 27.9% in the placebo group (*P* = .88) (Figure 4). Similar trends were observed in biologic-naive patients, except clinical response rates in the deucravacitinib 6-mg BID group were lower than those in the placebo group (Figure 2A). Efficacy rates achieved in the deucravacitinib 6-mg BID vs placebo group were 16.1% versus 0% for clinical remission, 29.0% versus 12.5% for clinical response, and 25.8% versus 12.5% for endoscopic response in biologic-exposed patients (Figure 2B).

#### 3.3.3. IM011-127

**Figure 5.**
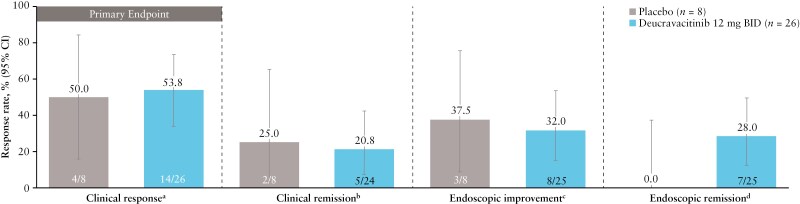
Week 12 efficacy in IM011-127 (intention-to-treat population; nonresponder imputation analysis). ^a^Defined as a reduction in the modified Mayo score of ≥2 points and ≥30% from baseline and a decrease in RBS score of ≥1 point or an absolute RBS of ≤1. The Mayo score (0–9 points) is the sum of the SFS, RBS, and MES (each individual subscore ranges from 0 to 3 points). ^b^Defined as an SFS of ≤1 with ≥1-point decrease from baseline, RBS of 0, and MES of ≤1 without friability. ^c^Defined as an MES of ≤1 without friability. ^d^Defined as an MES of 0. BID, twice daily; CI, confidence interval; MES, Mayo endoscopic subscore; RBS, rectal bleeding subscore; SFS, stool frequency subscore.

The study did not meet its primary endpoint of clinical response at week 12. This study was not powered for comparisons among the treatment groups. Clinical response rates were similar for deucravacitinib 12 mg BID and placebo (53.8% and 50.0%, respectively) (Figure 5). Similar trends were observed for clinical remission (20.8% and 25.0%) and endoscopic improvement (32.0% and 37.5%). Endoscopic remission, a more stringent endpoint, was achieved in 28.0% versus 0% of patients in the deucravacitinib 12 mg versus placebo, respectively (Figure 5).

### 3.4. Safety

Overall, AEs occurred in approximately 70%–81% in the deucravacitinib groups and 48%–75% in the placebo groups for all studies (Table 3). Acne and rash were the most common skin AEs reported in the deucravacitinib groups, the incidences of which were dose-dependent in LATTICE-CD (acne: 7.1% and 15.7%; rash: 6.0% and 4.8% in the deucravacitinib 3-mg and 6-mg BID groups, respectively), LATTICE-UC (acne: 9.2%; rash: 11.5% in deucravacitinib 6-mg BID group), and IM0111-127 (acne: 26.9%; rash: 19.2% in deucravacitinib 12-mg BID group); however, these events did not increase in severity with dose (ie, generally mild to moderate without an increased risk of treatment discontinuation). AEs leading to treatment discontinuation were observed in approximately 8%–15% in the deucravacitinib groups and 0%–7% in the placebo groups. The most common AE (≥2%) leading to discontinuation in LATTICE-CD was CD (deucravacitinib 3-mg and 6-mg BID: 6.0% and 3.6% vs placebo: 1.7%; Table S4). For LATTICE-UC, the most common AEs (≥2%) leading to discontinuation were UC exacerbation (deucravacitinib: 4.6% vs placebo: 2.4%) and COVID-19 pneumonia (3.4% vs 0%; Table S5). For IM011-127, there were 2 AEs that led to discontinuation in the deucravacitinib group (1 event of UC and *Bacteroides* bacteremia each) and none in the placebo group (Table S6). SAEs occurred in approximately 7%–15% in the deucravacitinib groups and 5%–13% in the placebo group (Table 3).

**Table 3.** Safety overview of LATTICE-CD, LATTICE-UC, and IM011-127.

	LATTICE-CD			LATTICE-UC		IM011-127	
	Placebo, *n* = 59	Deucravacitinib, 3 mg BID, *n* = 84	Deucravacitinib, 6 mg BID, *n* = 83	Placebo, *n* = 42	Deucravacitinib, 6 mg BID, *n* = 87	Placebo, *n* = 8	Deucravacitinib, 12 mg BID, *n* = 26
AEs, *n* (%)	38 (64.4)	66 (78.6)	66 (79.5)	20 (47.6)	61 (70.1)	6 (75.0)	21 (80.8)
SAEs, *n* (%)	6 (10.2)	8 (9.5)	4 (4.8)	2 (4.8)	8 (9.2)	1 (12.5)	4 (15.4)
AE leading to treatment discontinuation, *n* (%)	3 (5.1)	7 (8.3)	12 (14.5)	3 (7.1)	10 (11.5)	0 (0.0)	2 (7.7)
Deaths, *n* (%)	0 (0.0)	0 (0.0)	0 (0.0)	0 (0.0)	1 (1.1)^a^	0 (0.0)	0 (0.0)

Abbreviations: AE, adverse event; BID, twice daily; CD, Crohn’s disease; SAE, serious adverse event; UC, ulcerative colitis.

^a^This event was due to COVID-19–related pneumonia.

One patient experienced deep vein thrombosis on deucravacitinib 12 mg BID in the IM011-127 study. The patient was a 47-year-old female who had a fall resulting in injury to the left thigh and was eventually hospitalized. The patient received low-molecular-weight heparin and recovered while on deucravacitinib. The patient completed the induction period with no further complications. One death due to COVID-19-related pneumonia occurred in the deucravacitinib 6-mg group in LATTICE-UC.

Notably, no clinically meaningful laboratory abnormalities (ie, elevated creatinine phosphokinase or hyperlipidemia), no rhabdomyolysis, no major cardiac AEs, and no herpes zoster viral infections were reported with deucravacitinib at any evaluated dose.

Safety outcomes observed in LATTICE-CD, LATTICE-UC, and IM011-127 are described in detail in Tables S4–S6, respectively.

## 4. Discussion

Deucravacitinib, a selective TYK2 inhibitor,^17^ was evaluated in 3 phase 2 studies (LATTICE-CD, LATTICE-UC, and IM011-127) in patients with IBD. All 3 studies failed to meet their respective primary and secondary efficacy endpoints over 12 weeks of deucravacitinib treatment at various doses in patients with moderately to severely active CD or UC.

Overall, deucravacitinib was generally well tolerated and had no new safety signals.^23,31^ Skin treatment-emergent adverse events (TEAEs) were the most common reported TEAE, with the most frequently reported manifestations in the deucravacitinib groups in all the present trials being acne and rash, which were dose-dependent and mild to moderate in severity. This aligns with the dose-dependent increase in skin AEs observed in previous phase 1 and 2 studies in healthy subjects^32^ and in patients with psoriasis, respectively.^33^ Notably, deucravacitinib did not demonstrate any significant clinical abnormalities or significant increase in the AEs of interest associated with JAK 1, 2, 3 inhibitors, including selective second-generation JAK inhibitors.^17,34^ The safety profile of deucravacitinib in patients with IBD was consistent with the known safety profile observed in patients with psoriasis^23,31^

Reasons for the lack of significant clinical benefit observed with deucravacitinib versus placebo in these IBD trials are unclear. It is possible that TYK2 inhibition may not be an appropriate target for patients with IBD. Indeed, a phase 2 trial of VTX958, another TYK2 inhibitor, in patients with CD also did not meet its primary endpoint. Lack of efficacy may suggest that the dosage of deucravacitinib was insufficient for the treatment of UC; however, evaluated doses for deucravacitinib were higher than those required for psoriasis, which is aligned with preclinical studies.^22,35^ Additionally, the primary endpoint of IM011-127 was not achieved even though a high dose of deucravacitinib 12-mg BID was utilized.

Given the limitations of the present studies, findings should be interpreted with caution. However, deucravacitinib did demonstrate some clinical effect in patients with CD or UC. Notably, in LATTICE-CD, the difference in endoscopic response rates between the deucravacitinib 3-mg BID and placebo groups was statistically significantly different. More patients improved with deucravacitinib 3-mg BID versus deucravacitinib 6-mg BID and placebo in clinical response and PRO2 remission at week 12 in LATTICE-CD. Additionally, greater mean decrease from baseline in SES-CD was observed with deucravacitinib 3-mg and 6-mg BID compared to placebo at week 12. In LATTICE-UC, patients in the deucravacitinib 6-mg BID group achieved a higher rate of clinical response than those within the placebo group. In IM011-127, patients receiving deucravacitinib 12-mg BID achieved higher rates of endoscopic remission compared with placebo. The deucravacitinib 3-mg BID group in LATTICE-CD and the deucravacitinib 6-mg BID group in LATTICE-UC, respectively, induced better response in biologic-exposed patients than placebo. Collectively, these observations might suggest some effects of deucravacitinib on mucosal inflammation. As a result, our analysis of the first deucravacitinib studies in patients with IBD provided important information about TYK2 inhibition and IBD treatment.

These studies were limited by small sample sizes for treatment groups that may have been impacted by early study termination, especially for the deucravacitinib 6-mg group in the IM011-127 study. No interim analyses for studies terminated early were performed to determine delayed response. Study IM011-127 was not powered to make comparisons across treatment groups. Due to small sample sizes (in LATTICE-CD, 53, 69, and 61 patients in the placebo, deucravacitinib 3-mg BID, and deucravacitinib 6-mg BID groups completed the induction period, respectively; in LATTICE-UC, 35 and 69 patients in the placebo and deucravacitinib 6-mg BID groups completed the induction period, respectively; in IM011-127, 8 and 26 patients in the placebo and deucravacitinib 12-mg BID groups were randomized into the study, respectively), formal analyses regarding efficacy by corticosteroid use were not conducted to determine the impact of corticosteroid use on efficacy, especially within the placebo group. Although mechanistic work was completed in early clinical trials,^22,25^ our phase 2 studies did not further explore target engagement. However, a biomarker analysis for deucravacitinib from these studies is currently ongoing and will be reported in future publications.

## 5. Conclusions

Deucravacitinib at multiple doses did not demonstrate significant clinical benefit versus placebo in patients with moderately to severely active CD or UC. Deucravacitinib was safe and well tolerated.

## Supplementary Material

jjaf080_suppl_Supplementary_Material

## Data Availability

The Bristol Myers Squibb policy on data sharing may be found at https://www.bms.com/researchers-and-partners/independent-research/data-sharing-request-process.html.

## Previous presentation

Schreiber S, Powell N, Reider F, et al. Efficacy of deucravacitinib, an oral, selective, tyrosine kinase 2 inhibitor, in patients with moderately to severely active ulcerative colitis and prior exposure to biologic therapy: Subanalysis from the phase 2 LATTICE-UC study. Presented at ACG 2022; October 21–26, 2022; Charlotte, NC, USA.

Danese S, Panaccione R, D’Haens G, et al. Efficacy and safety of deucravacitinib, an oral, selective tyrosine kinase 2 inhibitor, in patients with moderately to severely active ulcerative colitis: 12-week results from the phase 2 LATTICE-UC study. Presented at DDW 2022; May 21–24, 2022; San Diego, CA, USA.

Danese S, Panaccione R, D’Haens, et al. Efficacy and safety of deucravacitinib, an oral, selective tyrosine kinase 2 inhibitor, in patients with moderately to severely active ulcerative colitis: 12-week results from the phase 2 LATTICE-UC study. Presented at ECCO 2022; Vienna, Austria.

Schreiber S, Powell N, Rieder F, et al. Efficacy of deucravacitinib, an oral, selective, tyrosine kinase 2 inhibitor, in patients with moderately to severely active ulcerative colitis and prior exposure to biologic therapy: subanalysis from the phase 2 LATTICE-UC study. Presented at UEGW 2022; October 8-11, 2022; Vienna, Austria.
