# Supplementary material for: Deucravacitinib in patients with inflammatory bowel disease: 12-week efficacy and safety results from 3 randomized phase 2 studies in Crohn’s disease and ulcerative colitis
Source: J Crohns Colitis. 2025 May 13;19(6):jjaf080. doi: 10.1093/ecco-jcc/jjaf080 (PMC12137900; doi:10.1093/ecco-jcc/jjaf080)

**SUPPLEMENTARY METHODS**

## Inclusion and Exclusion Criteria

*LATTICE-CD*

### Patients were excluded for any of the following: severe or fulminant colitis that was likely to require surgery or hospitalization; presence of a diagnosis of an alternative form of colitis (i.e., infectious, indeterminate, malignant); stoma, gastric or ileoanal pouch, previous proctocolectomy or total colectomy, abscess or suspected abscess, short-bowel syndrome, or history of bowel perforation; stool positive for Clostridioides difficile at screening; cancer or history of cancer; clinically relevant cardiac condition; inadequate response or loss of response to any medication that targets the same pathway as deucravacitinib (e.g., anti–interleukin 12 [IL-12]/23p40 antibodies, anti–IL-23p19 antibodies; however, patients who previously had an inadequate response or loss of response to ustekinumab were allowed to enroll toward the end of the study, when enrollment was at approximately 83%); or evidence of active or latent tuberculosis, hepatitis B or C, or HIV.

## Statistical Analysis

### LATTICE-CD

### With a two-sided chi-square test at a significance level of 0.025 and a calculated sample size of 240 patients (deucravacitinib 3-mg BID group, n = 90; deucravacitinib 6-mg BID group, n = 90; placebo group, n = 60), an 80% power was estimated to detect a treatment difference of 23% between active treatment (deucravacitinib 3 mg or 6 mg BID) and placebo for clinical remission during induction, and an 87% power was estimated to detect a treatment difference of 23% between active treatment and placebo for endoscopic response during induction.

#### To adjust for multiplicity of coprimary and secondary endpoints, alpha was split into two separate testing branches at a two-sided alpha value of 0.025 for deucravacitinib 3 mg BID compared with placebo and deucravacitinib 6 mg BID compared with placebo; nominal p values were provided for descriptive purposes.

### LATTICE-UC

### A planned sample size of 120 patients (deucravacitinib 6-mg BID group, n = 80; placebo group, n = 40) was estimated to provide approximately 82% power to detect a 15% treatment difference in clinical remission at Week 12 with a one-sided 0.1 level of significance.

*IM011-127*

### A planned sample size of 50 patients (deucravacitinib 12-mg BID group, n = 37; placebo group, n = 13) was not based on statistical power for comparison among treatment groups, but, rather, was selected to provide adequate precision for the estimation of efficacy rate for clinical response at Week 12 (~60%) and safety (98% probability of observing at least one occurrence of any AE with a 10% incidence or 90% probability of any AE with a 6% incidence rate).

**SUPPLEMENTARY TABLE 1** Efficacy endpoint definitions.

| **Endpoint** | **Definition** |
| --- | --- |
| *LATTICE-CD* | |
| Clinical remission | CDAI of <150 |
| Endoscopic response | ≥50% improvement from baseline on the SES-CD |
| Clinical response | Reduction from baseline in the CDAI of ≥100 points or a total CDAI of <150 |
| PRO2 remission | Average daily score for abdominal pain of ≤1, an average number of very soft (loose) or liquid (watery) stools (BSS type 6 or 7 only) of ≤3 on the PRO2, and both not worse than baseline |
| *LATTICE-UC* | |
| Clinical remission | SFS of ≤1 with a ≥1-point decrease from baseline, RBS of 0, and MES of ≤1 without friability |
| Clinical response | Reduction in modified Mayo score^a^ of ≥2 points and ≥30% from baseline and a decrease in the RBS of ≥1 point or an absolute RBS of ≤1 |
| Endoscopic response | MES of ≤1 |
| *IM011-127* | |
| Clinical response | Reduction in the modified Mayo score^a^ of ≥2 points and ≥30% from baseline and a decrease in the RBS of ≥1 point or an absolute RBS of ≤1 |
| Clinical remission | SFS of ≤1 with a ≥1-point decrease from baseline, RBS of 0, and MES of ≤1 without friability |
| Endoscopic improvement | MES of ≤1 without friability |
| Endoscopic remission | MES of 0 |

^a^The modified Mayo score (0–9 points) is the sum of the SFS, RBS, and MES (each individual subscore ranges from 0 to 3 points).

BSS, Bristol Stool Scale; CDAI, Crohn’s Disease Activity Index; MES, Mayo endoscopic score; PRO2, patient-reported outcome based on the stool frequency and abdominal pain components of the CDAI; RBS, rectal bleeding subscore; SES-CD, Simple Endoscopic Score for Crohn’s Disease; SFS, stool frequency subscore.

**SUPPLEMENTARY TABLE 2** Enrollment and randomization of patients by country in LATTICE-CD, LATTICE-UC, and IM011-127.

| **Country, n (%)** | **LATTICE-CD** | | **LATTICE-UC** | | **IM011-127** | |
| --- | --- | --- | --- | --- | --- | --- |
|  | **Enrolled**  **(*n* = 571)** | **Randomized**  **(*n* = 239)** | **Enrolled**  **(*n* = 228)** | **Randomized**  **(*n* = 131)** | **Enrolled**  **(*n* = 94)** | **Randomized**  **(*n* = 38)** |
| United States of America | 179 (31.3) | 66 (27.6) | 43 (18.9) | 24 (18.3) | 14 (14.9) | 5 (13.2) |
| Poland | 84 (14.7) | 37 (15.5) | 104 (45.6) | 61 (46.6) | 55 (58.5) | 17 (44.7) |
| Russia | 32 (5.6) | 15 (6.3) | 15 (6.6) | 8 (6.1) | 0 | 0 |
| Germany | 29 (5.1) | 11 (4.6) | 2 (0.9) | 1 (0.8) | 5 (5.3) | 3 (7.9) |
| Brazil | 27 (4.7) | 7 (2.9) | 0 | 0 | 0 | 0 |
| China | 26 (4.6) | 12 (5.0) | 0 | 0 | 0 | 0 |
| Hungary | 22 (3.9) | 8 (3.3) | 6 (2.6) | 4 (3.1) | 0 | 0 |
| Japan | 21 (3.7) | 20 (8.4) | 8 (3.5) | 6 (4.6) | 0 | 0 |
| United Kingdom of Great Britain and Northern Ireland | 19 (3.3) | 10 (4.2) | 8 (3.5) | 4 (3.1) | 5 (5.3) | 3 (7.9) |
| Australia | 15 (2.6) | 7 (2.9) | 7 (3.1) | 5 (3.8) | 3 (3.2) | 0 |
| Italy | 15 (2.6) | 9 (3.8) | 21 (9.2) | 13 (9.9) | 0 | 0 |
| Mexico | 14 (2.5) | 4 (1.7) | 0 | 0 | 0 | 0 |
| Canada | 14 (2.5) | 3 (1.3) | 0 | 0 | 6 (6.4) | 5 (13.2) |
| Spain | 12 (2.1) | 5 (2.1) | 0 | 0 | 0 | 0 |
| France | 11 (1.9) | 3 (1.3) | 1 (0.4) | 0 | 0 | 0 |
| Czech Republic | 10 (1.8) | 6 (2.5) | 2 (0.9) | 0 | 0 | 0 |
| Republic of Korea | 6 (1.1) | 1 (0.4) | 8 (3.5) | 3 (2.3) | 0 | 0 |
| Netherlands | 6 (1.1) | 2 (0.8) | 0 | 0 | 6 (6.4) | 5 (13.2) |
| Romania | 6 (1.1) | 3 (1.3) | 0 | 0 | 0 | 0 |
| Taiwan | 5 (0.9) | 4 (1.7) | 0 | 0 | 0 | 0 |
| Ireland | 5 (0.9) | 1 (0.4) | 0 | 0 | 0 | 0 |
| Belgium | 4 (0.7) | 2 (0.8) | 3 (1.3) | 2 (1.5) | 0 | 0 |
| Denmark | 3 (0.5) | 1 (0.4) | 0 | 0 | 0 | 0 |
| Israel | 3 (0.5) | 1 (0.4) | 0 | 0 | 0 | 0 |
| Portugal | 2 (0.4) | 1 (0.4) | 0 | 0 | 0 | 0 |
| Switzerland | 1 (0.2) | 0 | 0 | 0 | 0 | 0 |

**SUPPLEMENTARY TABLE 3** List of academic investigators and clinicians by country who recruited patients in LATTICE-CD, LATTICE-UC, and IM011-127.

|  | **Investigator Name** | **Country** |
| --- | --- | --- |
| LATTICE-CD | James Fon | Australia |
|  | Yoon-Kyo An | Australia |
|  | Stephan Brown | Australia |
|  | Lena Thin | Australia |
|  | Jane Andrews | Australia |
|  | Gregory Moore | Australia |
|  | Jean-Francois Rahier | Belgium |
|  | Elisabeth Macken | Belgium |
|  | Pieter Dewint | Belgium |
|  | Sandra Boratto | Brazil |
|  | Mittermayer Barreto Santiago | Brazil |
|  | Andre Castro Lyra | Brazil |
|  | Cyrla Zaltman | Brazil |
|  | Wilson Roberto Catapani | Brazil |
|  | Juliano de Oliveira | Brazil |
|  | Geni Cunrath | Brazil |
|  | Carlos De Magalhaes Francesconi | Brazil |
|  | Allen Lim | Canada |
|  | James Gray | Canada |
|  | Michael Gould | Canada |
|  | Yiqun Hu | China |
|  | Jiangbin Wang | China |
|  | Hongjie Zhang | China |
|  | Jie Zhong | China |
|  | Fei Liu | China |
|  | Weihong Sha | China |
|  | Xiang Gao | China |
|  | Deliang Liu | China |
|  | Tomas Vanasek | Czech Republic |
|  | Pavel Svoboda | Czech Republic |
|  | Jan Ulbrych | Czech Republic |
|  | Sylva Brtnikova | Czech Republic |
|  | Jan Fallingborg | Denmark |
|  | Romain ALTWEGG | France |
|  | Barbara BOURNET | France |
|  | Laurent ALRIC | France |
|  | Xavier Roblin | France |
|  | Stefan Schreiber | Germany |
|  | Axel Schweitzer | Germany |
|  | Jochen Klaus | Germany |
|  | Wolfgang Reindl | Germany |
|  | Arne Kandulski | Germany |
|  | Irina Blumenstein | Germany |
|  | Yvonne Doerffel | Germany |
|  | Lars Fechner | Germany |
|  | Mihaly Makara | Hungary |
|  | Gyula Horvat | Hungary |
|  | Marta Varga | Hungary |
|  | Tibor Szaloki | Hungary |
|  | Zsolt Tulassay | Hungary |
|  | Zoltan Gabor Szepes PI | Hungary |
|  | Akos Ilias | Hungary |
|  | Orlaith Kelly | Ireland |
|  | Gerard Clarke | Ireland |
|  | Irit Avni-Biron | Israel |
|  | Wisam Sbeit | Israel |
|  | Ehud Melzer | Israel |
|  | Mauro Mastronardi | Italy |
|  | Massimo Fantini | Italy |
|  | Edoardo Savarino | Italy |
|  | Francesco Luzza | Italy |
|  | Alessandro Armuzzi | Italy |
|  | Di Sabatino Antonio | Italy |
|  | Silvio Danese | Italy |
|  | Kaoru Yokoyama | Japan |
|  | Shinji Tanaka | Japan |
|  | Motohiro Esaki | Japan |
|  | Masayuki Saruta | Japan |
|  | Shigeki Bamba | Japan |
|  | Shinji Katsushima | Japan |
|  | Kenkei Hasatani | Japan |
|  | Ken Sugimoto | Japan |
|  | Yoh Ishiguro | Japan |
|  | Katsuyoshi Matsuoka | Japan |
|  | Shinichiro Shinzaki | Japan |
|  | Eiji Takeshita | Japan |
|  | Naoki Omiya | Japan |
|  | Masahiro Iwabuchi | Japan |
|  | Satoshi Motoya | Japan |
|  | Hyun Soo Kim | Korea, South |
|  | Jun Hwan Yoo | Korea, South |
|  | Sung Kook Kim | Korea, South |
|  | Yong Woon Shin | Korea, South |
|  | Claudio Gabriel Marmolejo Garcia | Mexico |
|  | Jorge Sanchez Beltran | Mexico |
|  | Graciela Torres | Mexico |
|  | Jose Leon Lopez Bucio | Mexico |
|  | Victoria del Carmen Padilla Rios | Mexico |
|  | Geert D'Haens | Netherlands |
|  | Marek Horynski | Poland |
|  | Grayna Rydzewska-Wyszkowska | Poland |
|  | Tomasz Romanczyk | Poland |
|  | Piotr Rozpondek | Poland |
|  | Michal Wiatr | Poland |
|  | Patryk Korga | Poland |
|  | Jaroslaw Kierkus | Poland |
|  | Mikolaj Krzyzanowski | Poland |
|  | Wit Danilkiewicz | Poland |
|  | Ewa Maecka-Panas | Poland |
|  | Radoslaw Kempinski | Poland |
|  | Wojciech Piotrowski | Poland |
|  | Maciej Gonciarz | Poland |
|  | Rute Cerqueira | Portugal |
|  | Adrian Goldis | Romania |
|  | Radu Bogdan Mateescu | Romania |
|  | Uspenskiy, Yuri | Russia |
|  | Ekaterina Valuyskikh | Russia |
|  | Marina, Osipenko | Russia |
|  | Natalya Voloshina | Russia |
|  | Irina Belova | Russia |
|  | Olga Reshetko | Russia |
|  | Konstantin Apartsin | Russia |
|  | Elena Ricart Gomez | Spain |
|  | Ana Alvarez Castro | Spain |
|  | Ana Gutierrez Casbas | Spain |
|  | Miguel Minguez Perez | Spain |
|  | Daniel Ceballos Santos | Spain |
|  | Montserrat Rivero Tirado | Spain |
|  | Fernando Bermejo San Jose | Spain |
|  | Frank Seibold | Switzerland |
|  | Chia-Hung Tu | Taiwan |
|  | Mithun Nagari | United Kingdom |
|  | Mohammad Nabil Quraishi | United Kingdom |
|  | Jonathan MacDonald | United Kingdom |
|  | Tim Raine | United Kingdom |
|  | Parakkal Deepak | United States |
|  | Maria Abreu | United States |
|  | Blanche Fung Liu | United States |
|  | Sarah Glover | United States |
|  | Nitin Gupta | United States |
|  | Scott Lee | United States |
|  | Francis Farraye | United States |
|  | Humberto Aguilar | United States |
|  | Nicholas Martinez | United States |
|  | Christopher Shepela | United States |
|  | Timothy Zisman | United States |
|  | Marc Schwartz | United States |
|  | Timothy Ritter | United States |
|  | Vrijendra Hoon | United States |
|  | George DuVall | United States |
|  | Lawrence Michael Weiss | United States |
|  | William Pandak | United States |
|  | Karlee Ausk | United States |
|  | Najm Soofi | United States |
|  | Kwabena Ayesu | United States |
|  | John Weber | United States |
|  | Harry Sarles | United States |
|  | Monika Fischer | United States |
|  | John Hanson | United States |
|  | Allan Coates | United States |
|  | Tawfik Chami | United States |
|  | Jeff Bullock | United States |
|  | Sumit Walia | United States |
|  | Philip Ginsburg | United States |
|  | Steven Polyak | United States |
|  | Alfred McNair | United States |
|  | Ronald Fogel | United States |
|  | Edward Tavel | United States |
|  | Michael Georgetson | United States |
|  | Curtis Freedland | United States |
|  | Lon Lynn | United States |
|  | Ravi Moparty | United States |
|  | Bruce Salzberg | United States |
| LATTICE-UC | Vrijendra Hoon | United States |
|  | Francesco Luzza | Italy |
|  | Ravi Moparty | United States |
|  | Bruce Salzberg | United States |
|  | Michal Wiatr | Poland |
|  | Tomasz Romanczyk | Poland |
|  | Olga Reshetko | Russia |
|  | Edoardo Savarino | Italy |
|  | Humberto Aguilar | United States |
|  | Marina Osipenko | Russia |
|  | Chang Hwan Choi | Korea, South |
|  | Sung Kook Kim | Korea, South |
|  | Gyula Horvat | Hungary |
|  | Mihaly Makara | Hungary |
|  | Antonio Di Sabatino | Italy |
|  | Michat Talarek | Poland |
|  | Piotr Gietka | Poland |
|  | Tim Raine | United Kingdom |
|  | Walter Fries | Italy |
|  | Silvio Danese | Italy |
|  | YooJin Lee | Korea, South |
|  | Robert Atkinson | United Kingdom |
|  | Jerzy Rozciecha | Poland |
|  | Dirk Staessen | Belgium |
|  | Dariusz Kleczkowski | Poland |
|  | Zsolt Tulassay | Hungary |
|  | Ana Elosegui | United States |
|  | Wojciech Piotrowski | Poland |
|  | Giovanni Monteleone | Italy |
|  | Louis Korman | United States |
|  | Curtis Freedland | United States |
|  | Blanche Fung Liu | United States |
|  | Robert Petryka | Poland |
|  | Beata Gawdis-Wojnarska | Poland |
|  | Martin Peterka | Czech Republic |
|  | Ingolf Schiefke | Germany |
|  | KyeongOk Kim | Korea, South |
|  | Vinciane Muls | Belgium |
|  | Toshimitsu Fujii | Japan |
|  | Motohiro Esaki | Japan |
|  | Stefan Schreiber | Germany |
|  | Reme Mountifield | Australia |
|  | Gerald Holtmann | Australia |
|  | Nicholas Martinez | United States |
|  | Erin Forster | United States |
|  | Keiichi Mitsuyama | Japan |
|  | Mohammed Al-Ansari | Australia |
|  | Kaoru Yokoyama | Japan |
|  | Tomohiro Kudo | Japan |
|  | Maged Ghali | United States |
|  | Jaroslaw Kierkus | Poland |
|  | Patryk Korga | Poland |
|  | Natalya Voloshina | Russia |
|  | Evgeny Chesnokov | Russia |
|  | Beata Neneman | Poland |
|  | Piotr Radziszewski | Poland |
|  | Harry Sarles | United States |
|  | Mikolaj Krzyzanowski | Poland |
|  | Ewa Malecka-Panas | Poland |
|  | Yong Woon Shin | Korea, South |
|  | Lena Thin | Australia |
|  | Jeff Bullock | United States |
|  | Miles Sparrow | Australia |
|  | Laurent Alric | France |
|  | George DuVall | United States |
|  | Jonathon Chapman | United States |
|  | Jason Etzel | United States |
| IM011-127 | Edward Shelton | Australia |
|  | Simon Ghaly | Australia |
|  | Stefan Schreiber | Germany |
|  | Jesse Siffledeen | Canada |
|  | Geert D'Haens | Netherlands |
|  | Esther Torres | USA |
|  | Benjamin Cohen | USA |
|  | Humberto Aguilar | USA |
|  | Sebastian Zeissig | Germany |
|  | Sumit Walia | USA |

**SUPPLEMENTARY TABLE 4** Safety assessments during in the induction period of LATTICE-CD in patients who received ≥1 treatment dose.

|  | **Placebo *n* = 59** | **Deucravacitinib**  **3 mg BID *n* = 84** | **Deucravacitinib**  **6 mg BID *n* = 83** |
| --- | --- | --- | --- |
| Most frequent AEs (≥5% in either of the deucravacitinib groups^a^), *n* (%) | | | |
| Acne | 1 (1.7) | 6 (7.1) | 13 (15.7) |
| Crohn’s disease | 7 (11.9) | 7 (8.3) | 10 (12.0) |
| Upper respiratory tract infection | 1 (1.7) | 7 (8.3) | 3 (3.6) |
| Headache | 3 (5.1) | 6 (7.1) | 4 (4.8) |
| Aphthous ulcer | 1 (1.7) | 2 (2.4) | 7 (8.4) |
| Mouth ulceration | 0 (0.0) | 5 (6.0) | 4 (4.8) |
| Rash | 2 (3.4) | 5 (6.0) | 4 (4.8) |
| COVID-19 | 5 (8.5) | 3 (3.6) | 6 (7.2) |
| Pyrexia | 4 (6.8) | 1 (1.2) | 7 (8.4) |
| Nasopharyngitis | 1 (1.7) | 3 (3.6) | 5 (6.0) |
| SAEs, *n* (%) | | | |
| Crohn’s disease | 3 (5.1) | 4 (4.8) | 2 (2.4) |
| Gastritis | 0 (0.0) | 1 (1.2) | 0 (0.0) |
| Ileal perforation | 0 (0.0) | 1 (1.2) | 0 (0.0) |
| Necrotizing stomatitis | 0 (0.0) | 1 (1.2) | 0 (0.0) |
| Acute hepatic failure | 0 (0.0) | 0 (0.0) | 1 (1.2) |
| Diabetic foot infection | 0 (0.0) | 1 (1.2) | 0 (0.0) |
| Norovirus test positive | 0 (0.0) | 1 (1.2) | 0 (0.0) |
| Hypophosphatemia | 0 (0.0) | 0 (0.0) | 1 (1.2) |
| Tracheal stenosis | 0 (0.0) | 0 (0.0) | 1 (1.2) |
| Hematoma | 0 (0.0) | 0 (0.0) | 1 (1.2) |
| Depression | 1 (1.7) | 0 (0.0) | 0 (0.0) |
| Subileus | 1 (1.7) | 0 (0.0) | 0 (0.0) |
| Abdominal abscess | 1 (1.7) | 0 (0.0) | 0 (0.0) |
| Anal abscess | 1 (1.7) | 0 (0.0) | 0 (0.0) |
| Lung abscess | 1 (1.7) | 0 (0.0) | 0 (0.0) |
| AEs leading to treatment discontinuation, *n* (%) | | | |
| Crohn’s disease | 1 (1.7) | 5 (6.0) | 3 (3.6) |
| Anemia | 0 (0.0) | 0 (0.0) | 2 (2.4) |
| Hypersensitivity | 0 (0.0) | 0 (0.0) | 2 (2.4) |
| Lymphopenia | 0 (0.0) | 1 (1.2) | 0 (0.0) |
| Acne | 0 (0.0) | 0 (0.0) | 1 (1.2) |
| Rash | 0 (0.0) | 0 (0.0) | 1 (1.2) |
| Acute hepatic failure | 0 (0.0) | 0 (0.0) | 1 (1.2) |
| Hemoglobin decreased | 0 (0.0) | 0 (0.0) | 1 (1.2) |
| Rheumatoid arthritis | 0 (0.0) | 1 (1.2) | 0 (0.0) |
| Stomatitis | 0 (0.0) | 0 (0.0) | 1 (1.2) |
| Anal abscess | 1 (1.7) | 0 (0.0) | 0 (0.0) |
| Subileus | 1 (1.7) | 0 (0.0) | 0 (0.0) |

^a^Deucravacitinib 12 mg QD AEs are not shown.

AE, adverse event, BID, twice daily; QD, once daily; SAE, serious adverse event.

**SUPPLEMENTARY TABLE 5** Safety assessments during the induction period of LATTICE-UC in patients who received ≥1 treatment dose.

|  | **Placebo *n* = 42** | **Deucravacitinib**  **6 mg BID *n* = 87** |
| --- | --- | --- |
| Most frequent AEs (≥5% in the deucravacitinib group), *n* (%) | | |
| Rash | 0 (0.0) | 10 (11.5) |
| Acne | 1 (2.4) | 8 (9.2) |
| Ulcerative colitis | 2 (4.8) | 6 (6.9) |
| SAEs, *n* (%) | | |
| COVID-19 pneumonia | 0 (0.0) | 3 (3.4) |
| COVID-19 | 0 (0.0) | 1 (1.1) |
| Anal abscess | 0 (0.0) | 1 (1.1) |
| Ulcerative colitis | 2 (4.8) | 2 (2.3) |
| Constipation | 0 (0.0) | 1 (1.1) |
| Anemia | 0 (0.0) | 1 (1.1) |
| AEs leading to treatment discontinuation, *n* (%) | | |
| Ulcerative colitis | 1 (2.4) | 4 (4.6) |
| COVID-19 pneumonia | 0 (0.0) | 3 (3.4) |
| COVID-19 | 0 (0.0) | 1 (1.1) |
| Anemia | 1 (2.4) | 1 (1.1) |
| Upper abdominal pain | 0 (0.0) | 1 (1.1) |
| Rash | 0 (0.0) | 1 (1.1) |
| Positive hepatitis B DNA assay | 1 (2.4) | 0 (0.0) |

AE, adverse event, BID, twice daily; SAE, serious adverse event.

**SUPPLEMENTARY TABLE 6** Safety assessments during in the induction period of IM011-127 in patients who received ≥1 treatment dose.

|  | **Placebo *n* = 8** | **Deucravacitinib**  **12 mg BID *n* = 26** |
| --- | --- | --- |
| Most frequent AEs (≥5% in the deucravacitinib group^a^), *n* (%) | 6 (75.0) | 21 (80.8) |
| Acne | 1 (12.5) | 7 (26.9) |
| Rash | 0 | 5 (19.2) |
| Nasopharyngitis | 1 (12.5) | 3 (11.5) |
| Pyrexia | 0 | 3 (11.5) |
| Aphthous ulcer | 0 | 2 (7.7) |
| Colitis ulcerative | 2 (25.0) | 2 (7.7) |
| Cough | 0 | 2 (7.7) |
| Mouth ulceration | 0 | 2 (7.7) |
| Pharyngitis | 0 | 2 (7.7) |
| Upper respiratory tract infection | 0 | 2 (7.7) |
| SAEs, *n* (%) |  |  |
| Ulcerative colitis | 1 (12.5) | 2 (7.7) |
| *Bacteroides* bacteriemia | 0 (0.0) | 1 (3.8) |
| Nephrolithiasis | 0 (0.0) | 1 (3.8) |
| Deep vein thrombosis | 0 (0.0) | 1 (3.8)^b^ |
| AEs leading to treatment discontinuation, *n* (%) |  |  |
| Ulcerative colitis | 0 (0.0) | 1 (3.8) |
| *Bacteroides* bacteriemia | 0 (0.0) | 1 (3.8) |

^a^Deucravacitinib 6 mg BID AEs are not shown.

^b^Patient was a 47-year-old female who had a fall resulting in injury to her left thigh and was eventually hospitalized. The patient was treated with low–molecular-weight heparin and recovered while on deucravacitinib. The patient completed the induction period with no further complications.

AE, adverse event, BID, twice daily; SAE, serious adverse event.

**Supplementary Figure 1.** Coprimary endpoints at Week 12 by prior biologic exposure in LATTICE-CD: [A] biologic-naive and [B] biologic-exposed patients (intention-to-treat population). ^a^Defined as CDAI of <150. ^b^Defined as ≥50% improvement from baseline in SES-CD. BID, twice daily; CI, confidence interval; CDAI, Crohn’s Disease Activity Index; SES-CD, Simple Endoscopic Score for Crohn’s Disease.


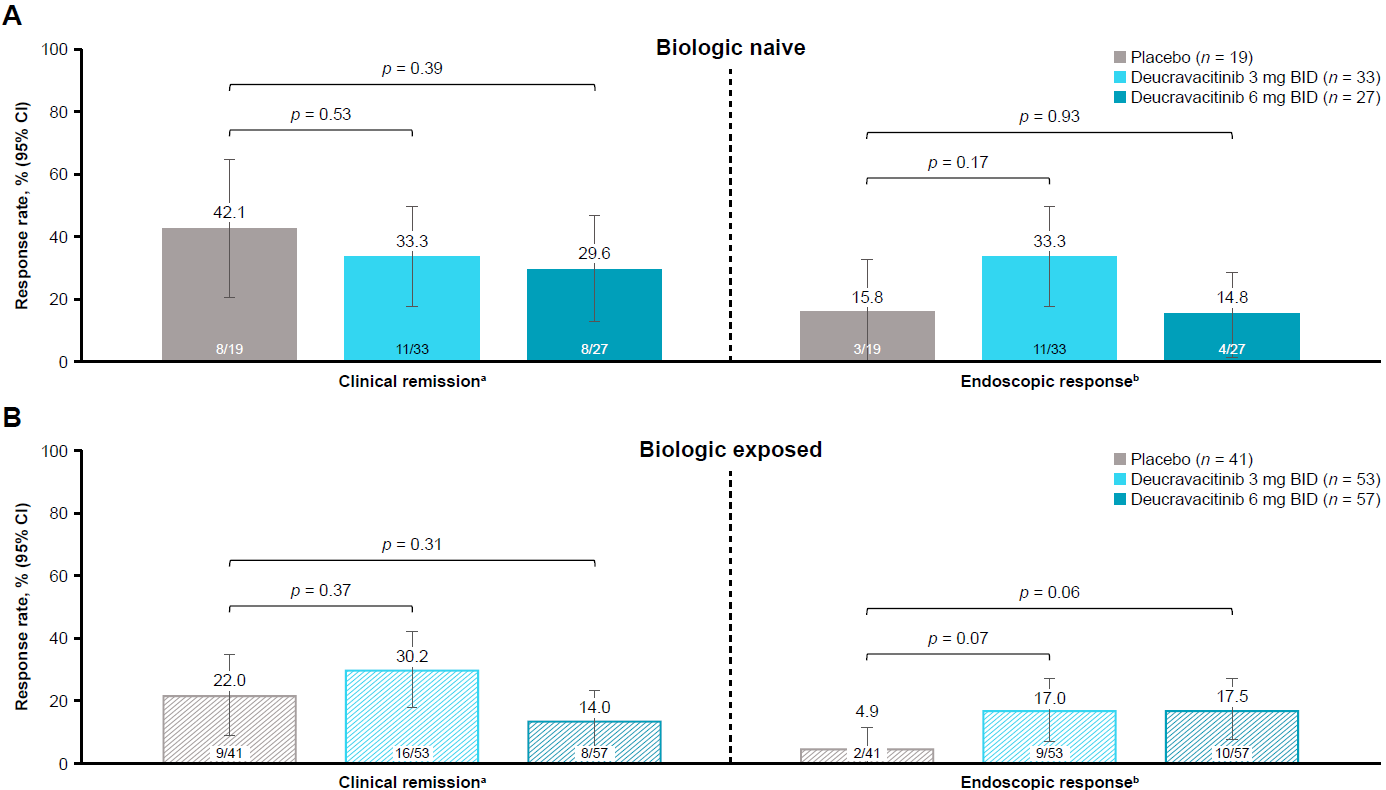


**Supplementary Figure 2.** Week 12 efficacy in LATTICE-UC by prior biologic use: [A] biologic-naive and [B] biologic-exposed (≥1 biologic) patients (intention-to-treat population). ^a^Defined as a stool frequency subscore (SFS) of ≤1 with ≥1-point decrease from baseline, rectal bleeding subscore (RBS) of 0, and Mayo endoscopic subscore (MES) of ≤1 without friability. ^b^Reduction in the modified Mayo score of ≥2 points and ≥30% from baseline and a decrease in the RBS of ≥1 point or an absolute RBS of ≤1. The Mayo score (0–9 points) is the sum of the SFS, RBS, and MES (each individual subscore ranges from 0 to 3 points). ^c^Defined as an MES of ≤1. BID, twice daily; CI, confidence interval.


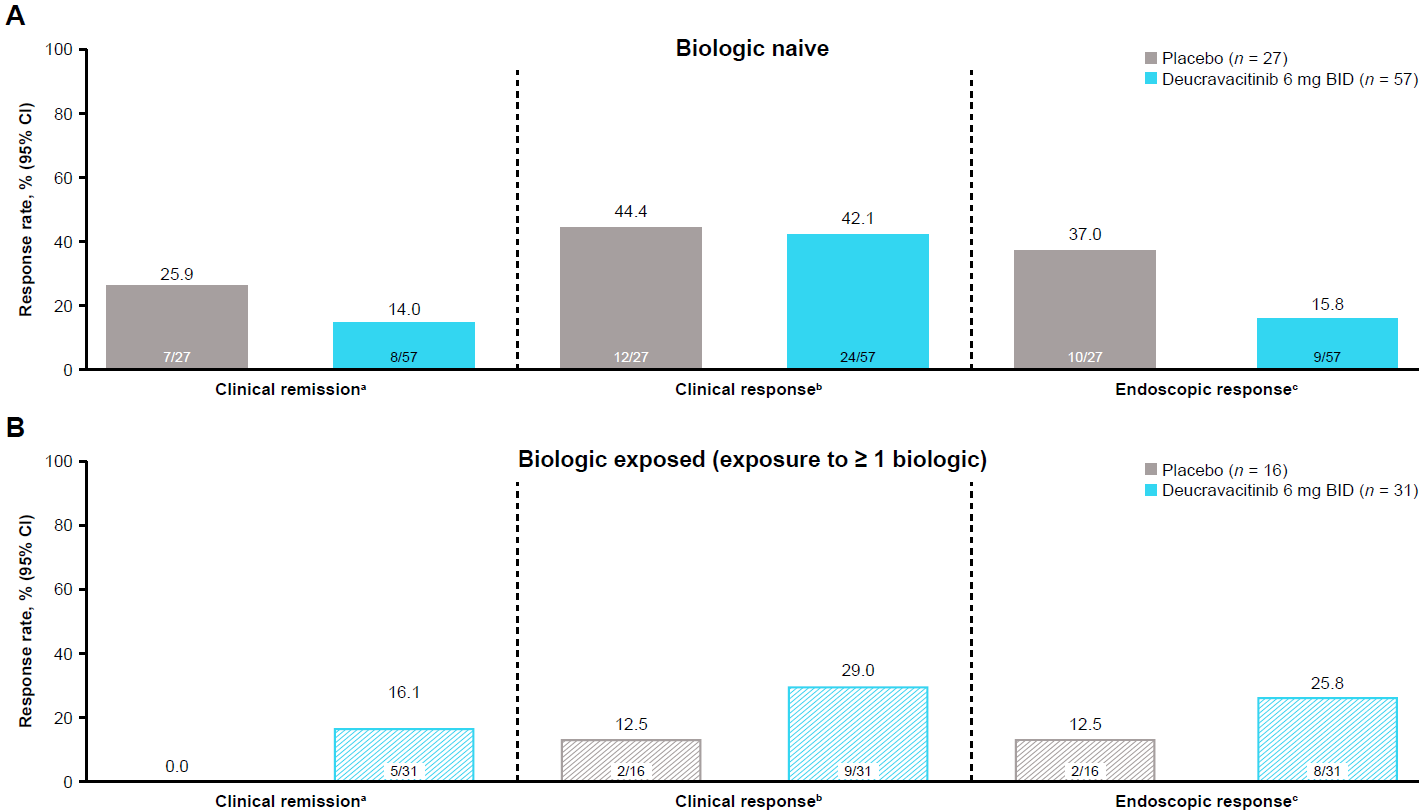

Supplement: jjaf080_suppl_Supplementary_Material [file jjaf080_suppl_supplementary_material.docx]
